# Beyond GLM: Inter-Subject Variability as a Complementary Approach to Detect Longitudinal Changes in Emotion Processing in Multiple Sclerosis

**DOI:** 10.3390/jimaging12050210

**Published:** 2026-05-15

**Authors:** Alice Pirastru, Valeria Blasi, Diego Michael Cacciatore, Marco Rovaris, Elena Toselli, Francesco Pagnini, Cesare Cavalera, Fabrizio Esposito, Giuseppe Baselli, Francesca Baglio

**Affiliations:** 1 IRCCS Fondazione Don Carlo Gnocchi ETS, Via Capecelatro 66, 20148 Milan, Italy; apirastru@dongnocchi.it (A.P.); dcacciatore@dongnocchi.it (D.M.C.); mrovaris@dongnocchi.it (M.R.); etoselli@dongnocchi.it (E.T.); fbaglio@dongnocchi.it (F.B.); 2 Department of Psychology, Università Cattolica del Sacro Cuore, 20123 Milan, Italy; francesco.pagnini@unicatt.it (F.P.); cesarem.cavalera@unicatt.it (C.C.); 3 Department of Advanced Medical and Surgical Sciences, University of Campania “Luigi Vanvitelli”, 80138 Naples, Italy; fabrizio.esposito@unicampania.it; 4 Department of Electronics, Information, and Bioengineering, Politecnico di Milano, 20123 Milan, Italy; giuseppe.baselli@polimi.it

**Keywords:** depression, EMDR, emotion stimulation task-fMRI, inter-subject variability, multiple sclerosis, rehabilitation

## Abstract

Understanding how to reliably capture neural changes induced by treatments in neurological patients remains a major methodological challenge. This issue is particularly evident in the emotional domain—frequently impaired in conditions such as multiple sclerosis (MS) and a key target of rehabilitation—yet not limited to it. Longitudinal neuroimaging studies predominantly rely on group-level analyses (e.g., General Linear Model, GLM), which assume inter-subject homogeneity and treat inter-subject variability (ISV) as noise. Such assumptions may obscure treatment-related neuroplastic changes, especially in domains like emotion processing, where neural responses are intrinsically variable and highly individualized in clinical populations. This study investigates whether modeling ISV can better capture treatment-related neural changes, using emotion-focused rehabilitation as a representative case. We compared GLM with threshold-weighted overlap maps (${OM}_{th-w}$), which quantify spatial consistency across individuals. Thirty healthy controls (HCs) and thirteen people with MS (pwMS) undergoing EMDR for depression performed an emotional fMRI task (pwMS pre/post-treatment). GLM revealed no longitudinal effects, whereas ${OM}_{th-w}$ showed reduced variability in pwMS after treatment, alongside decreased depressive symptoms (*p* < 0.001). These findings highlight the value of variability-based approaches as a complementary framework to conventional GLM analyses for detecting treatment-related neuroplasticity in neurological populations.

## 1. Introduction

Understanding how to reliably capture neural changes induced by rehabilitation in neurological patients remains a major methodological challenge. Although neuroimaging is increasingly used to assess treatment effects, identifying consistent markers of neuroplasticity across individuals remains difficult, particularly in longitudinal designs that rely on group-level approaches and may fail to capture individual trajectories or heterogeneous patterns of neural reorganization. This challenge is especially evident in clinically relevant and neurologically complex domains such as emotion processing. Emotion processing, pivotal for social abilities, is reportedly impaired in different neurological and neurodegenerative conditions, leading to potential behavioral disorders such as depression [1]. For this reason, it is often targeted by non-pharmacological interventions such as rehabilitation, and effective tools are needed to help understand the neural mechanisms underlying the deficit and restoration of social abilities. This is particularly complex since the emotional domain is characterized by high variability at the intra- and inter-subject levels, both in terms of behavioral [2] and neural response [3,4,5].

The concept of inter-individual or between-subject variability is linked to both anatomic-functional differences [6,7] and diverse neural strategies [8] employed for the execution of the same task, possibly arising from previous experiences and environmental factors. In neuroimaging, this variability is reflected in heterogeneous brain activations, and it is observed even among healthy individuals, due to different brain morphology and functional organization (i.e., intrinsic variability). Moreover, a strategic variability, namely the accomplishment of the same task in multiple ways, gains a major weight in neural degeneracy and individual compensation strategies, which is reportedly the prevalent source of inter-individual variability. At the neural level, the strategic variability results in the engagement of diverse brain areas and circuits in response to the same task, as shown for a variety of brain functions and tasks in fMRI, such as visual processing [3], language processing [9,10,11], memory [3,12,13,14], reading [15] and emotion processing [3].

The effectiveness of the inter-subject variability assessment, specifically regarding subjects’ ability to switch between different strategies relying on different neural circuits [16], has been highlighted as crucial for diagnosis [17], rehabilitation [18], and the prediction of single-subject activation [19].

However, neuroimaging studies typically assume temporal and spatial similarities of brain function across individuals, relying on standard group-level analyses, seeking localized average neural activation in response to the same stimulus. Specifically, fMRI analyses are most commonly based on the general linear model (GLM), a mass-univariate framework that estimates voxel-wise task-related effects by fitting the BOLD signal to a predefined design matrix and testing for condition-specific contrasts [20,21]. While highly effective in detecting group-level effects, this approach may fail to capture meaningful between-subject differences: the individual factor is overlooked in favor of collective effects, and the inter-individual variability is treated as noise or measurement error [16]. Furthermore, the estimated group effect might not be representative as the main effect for any of the individuals included in the sample, especially when considering atypical patterns of activation characterizing clinical populations in which the heterogeneity constitutes valuable information to reveal subject-specific neuroplasticity mechanisms.

To address this limitation, alternative approaches, such as threshold-weighted overlap maps (${OM}_{th-w}$), have been proposed to explicitly quantify inter-subject variability [22]. Rather than focusing on average activation, these maps estimate the spatial consistency of neural responses by quantifying the proportion of subjects showing significant activation within each voxel or region across multiple statistical thresholds, thereby providing an intuitive measure of shared versus heterogeneous activation patterns. This approach offers a complementary perspective to standard GLM analyses, particularly in longitudinal and clinical contexts where individual variability is expected.

In this study, we aimed to assess the inter-subject variability and verify the relevance of this approach in detecting neuroplasticity mechanisms in a longitudinal experimental setting involving people with multiple sclerosis (pwMS) undergoing an “eye movement desensitization and reprocessing” (EMDR) treatment to address depressive symptoms [23]. Specifically, ${OM}_{th-w}$ maps were compared to standard general linear model (GLM) group analyses derived from the same dataset to provide a complementary and more comprehensive analysis exploring longitudinal effects. To this end, an emotion stimulation fMRI task [24] was employed to explore neural activation in pwMS before and after the EMDR treatment in a cohort of healthy subjects.

## 2. Materials and Methods

### 2.1. Participants Inclusion Criteria

Thirty healthy volunteers were enrolled in the study and underwent two separate MRI acquisitions within a 3- to 5-month inter-scan interval. We considered the absence of neurological conditions and neuropsychiatric disorders as assessed by clinical interviews as inclusion criteria. The presence of any contraindication to perform an MRI examination (e.g., claustrophobia, presence of metallic prosthetics, MRI unsafe devices, etc.) represented an exclusion criterion. Thirteen pwMS, showing depressive symptoms as MS-associated comorbidity, were also included in this work as a case study [23] according to the following inclusion criteria: MS diagnosis [25], ability to communicate and understand tasks, verified depressive symptoms as assessed with depression scales (Hospitalized Anxiety and Depression Scale, HADS, and the Hamilton Depression Rating Scale, HDRS), stable pharmacological treatment, absence of severe neuropsychological impairment, and no ongoing psychotherapeutic treatment for depressive symptoms.

pwMS underwent two separate MRI acquisitions before and after an EMDR rehabilitative treatment [23].

This study was approved by the Institutional Research Ethical Committee of IRCCS Fondazione Don Gnocchi Ethical Committee on 16 April 2019 (Protocol Number: 13/2019/CE_FdG/FC/SA). All the subjects signed a written informed consent.

### 2.2. MRI Data Acquisition and Preprocessing

The MRI scan protocol was acquired on a 3 T Siemens Prisma Scanner (Erlangen, Germany) equipped with a 64-channel head/neck coil. The protocol included 3 acquisition sequences: (1) a structural sequence, namely T1-3D magnetization prepared rapid acquisition with gradient-echo (MPRAGE) sequence with repetition time (TR) = 2300 ms, echo time (TE) = 3.1 ms, isotropic resolution = 0.8 × 0.8 × 0.8 mm^3^, 224 slices, used as an anatomical reference; (2) a sagittal fluid-attenuated inversion recovery (FLAIR) sequence (TR = 5000 ms, TE = 394 ms, resolution = 0.8 × 0.8 × 1 mm^3^, acquisition matrix = 288 × 320, 176 slices), acquired to exclude gross brain abnormalities and to segment MS lesions; and (3) an accelerated GE functional sequence with TR = 2000 ms, TE = 30 ms, resolution 3 × 3 × 3 mm^3^, multi-slice acceleration factor = 2, 52 slices, 330 measurements, which was acquired during the task administration and consisted of an emotion stimulation paradigm, using visual stimuli selected from the International Affective Picture System (IAPS) database [26]. The details of the task and its acquisition are reported in our previous paper [24]. The acquisition was repeated twice, during which one of the two parallel forms of the fMRI task was randomly administered to account for stimulus habituation [24].

Task stimuli were delivered using E-Prime 3.0 (Psychology Software Tools, https://pstnet.com/products/e-prime/ (accessed on 10 May 2026)) using a NordicNeuroLab system (https://www.nordicneurolab.com/) comprising an “in-room viewing device” with an MR-compatible display located at the end of the gantry and a mirror placed on the head coil. The stimuli administration was synchronized with the MR acquisition using a dedicated device (SyncBox, NordicNeuroLab, Bergen, Norway).

The structural MPRAGE images were preprocessed using the FMRIB Software Library v6.0.7.17 (FSL, https://fsl.fmrib.ox.ac.uk/fsl/docs/ (accessed on 10 May 2026)) according to the following steps: bias field correction [27], brain extraction [28,29], and lesion filling [30] (for images acquired from pwMS).

The white matter hyperintensities for pwMS were segmented by an experienced operator on the FLAIR images using Jim (v6.0_35), a semiautomatic tool (https://www.xinapse.com/). Lesion masks were derived from the segmentation and used to perform the lesion-filling correction on the MPRAGE images. The functional volumes were preprocessed using the statistical parametric mapping toolbox (SPM12 https://www.fil.ion.ucl.ac.uk/spm/ (accessed on 10 May 2026)), following standard steps consisting of motion correction and realignment, co-registration with anatomical MPRAGE, normalization to the standard MNI template and spatial smoothing considering an 8 FWHM kernel. None of the subjects included in the study exceeded the limit of 2 mm in translation and/or 2° in rotation in head motion. All the preprocessing steps were visually inspected by experienced operators (A.P.; V.B.).

The first-level analysis was conducted using the GLM to fit the statistical model to the BOLD response. The 4 conditions included in the task design were considered as regressors of interest. The six motion parameters were included instead as nuisance regressors. At the subject level, two contrasts were derived comparing either positive or negative stimuli conditions versus the neutral stimuli condition. Individual unthresholded t-statistic maps (t-maps) were generated to map the subject-specific fixed effects to be used to derive threshold-weighted overlap maps (${OM}_{th-w}$, see Section 2.4).

The second-level analyses, either based on GLM statistics or relying on (${OM}_{th-w}$), were conducted by restricting the neural activation patterns to 16 bilateral regions of interest (ROIs) chosen according to areas that resulted significantly activated during emotion processing as shown in our previous study employing the same experimental settings [24]. Specifically, the (bilateral) ROIs were: inferior frontal cortex pars opercularis, pars orbitalis, pars triangularis, middle superior frontal cortex, insula, inferior parietal cortex, precuneus, fusiform cortex, hippocampus, para-hippocampal cortex, middle temporal cortex, cuneus cortices, inferior occipital cortex, middle occipital cortex, amygdala, and thalamus. The ROI has been derived according to the automated anatomical labeling 3 (AAL3) atlas [31], using the AAL3 toolbox embedded in SPM12.

### 2.3. GLM Random Effect Group Analysis

In second-level group analysis, all subjects were treated as random effects, and single-group activation maps for positive and negative emotions were obtained by means of one-sample *t*-tests. The observed neural activations were considered statistically significant for $p_{FWE}$ < 0.05, applying the family-wise error (FWE) correction for multiple comparisons to account for false positives. The single-group activation maps were derived separately for the two acquisitions within healthy participants and pwMS groups.

Finally, the activation maps obtained for pwMS pre- and post-rehabilitation treatment were compared using a paired *t*-test for both positive and negative contrasts.

### 2.4. Threshold-Weighted Overlap Maps

At the group level, ${OM}_{th-w}$ were derived to explore inter-subject variability according to [22].

The normalized t-maps derived at the single-subject level, both for the positive and negative emotional valence contrasts, were used to derive overlap maps (${OM}_{v,th}$) at 3 selected statistical thresholds (th), namely $p_{unc}$_c_ < 0.05, $p_{unc}$ < 0.01, and $p_{unc}$ < 0.001 (corresponding to T-values equal to 1.65, 2.34 and 3.12 respectively) according to [22]:

$${OM}_{v,th}=\frac{1}{S}\cdot \sum_{n=1}^{S}\left\{\begin{array}{c} 0,\;T_{n,v}\;<th\\ 1,\;T_{n,v}\;\geq th \end{array}\right.$$

where S = subjects, and v = voxels.

${OM}_{v,th}$, namely ${OM}_{v,p_{unc}}$ < 0.05, ${OM}_{v,p_{unc}}$ < 0.01, ${OM}_{v,p_{unc}}$ < 0.001, obtained as detailed above, represent spatial consistency maps quantifying the percentage of subjects sharing a neural activation within a specific location for the given threshold. To assess the consistency independently of the statistical threshold, the 3 maps were then combined according to

$$\left\{\begin{array}{c} {OM}_{th-w}=\int_{T_{min}}^{T_{max}}W_{th}\cdot {OM}_{v,th}\cdot dth\\ W_{th}=\frac{2}{T_{max}^{2}-T_{min}^{2}}\;\cdot th\; \end{array}\right.$$

A linear weighting was adopted, yielding a maximal consistency equal to 1 (area of the histogram) and enabling a qualitative assessment [32] of the consistency, as typically done in psychology (e.g., OM < 0.4: poor, 0.4 < OM < 0.6: fair, 0.6 ≤ OM ≤ 0.75: good, OM > 0.75: excellent). The integral was then discretized according to

$${OM}_{th-w}\approx \;\frac{{OM}_{v,p_{unc}<0.05}\cdot W_{p_{unc}<0.05}}{2}\cdot \left({th}_{{@p}_{unc}<0.05}\right)+\frac{{OM}_{v,p_{unc}<0.05}\cdot W_{p_{unc}<0.05}+{OM}_{v,p_{unc}<0.01}\cdot W_{p_{unc}<0.01}}{2}\;\cdot \left({th}_{{@p}_{unc}<0.01}-{th}_{{@p}_{unc}<0.05}\right)+\frac{{OM}_{v,p_{unc}<0.001}\cdot W_{p_{unc}<0.001}+{OM}_{v,p_{unc}<0.01}\cdot W_{p_{unc}<0.01}}{2}\;\cdot \left({th}_{{@p}_{unc}<0.001}-{th}_{{@p}_{unc}<0.01}\right)\;\;\;\;\;\;\;\;\;\;\;\;\;\;\;\;\;\;\;\;\;$$

**Figure 1:**
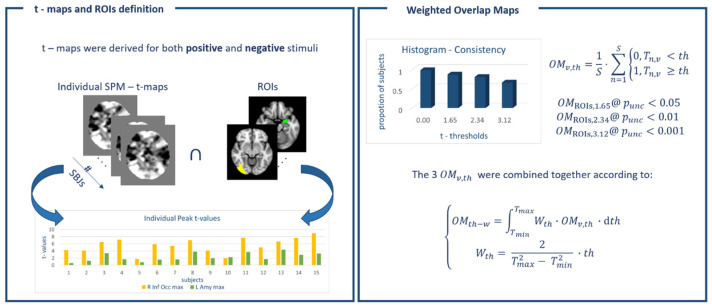
Outline of the analysis pipeline employed to derive threshold-weighted overlap maps (${OM}_{th-w}$) according to the method proposed by [22].

Given the 3 statistical thresholds chosen above (T@_punc<0.05_ = 1.65, T@_punc<0.01_ = 2.34 and T@_punc<0.001_ = 3.12), the weights will respectively assume the values of $W_{p_{unc}<0.05}=0.339,\;W_{p_{unc}<0.01}=0.481$ and $W_{p_{unc}<0.001}=\;0.641$. The steps employed to derive the ${OM}_{th-w}$ are summarized in Figure 1.

### 2.5. Statistical Analysis of ${OM}_{th-w}$-Derived Measures

Statistical comparisons were performed on overlap-derived summary measures to assess differences across groups, conditions and time points. Given that ${OM}_{th-w}$ represent group-level spatial consistency indices and do not provide subject-specific values, statistical inference was conducted at the level of predefined ROIs.

For each ROI, mean and peak overlap values were extracted separately for each condition (positive and negative emotional valence) and each time point (i.e., pre- vs. post-treatment in pwMS and 1st vs. 2nd scan in HCs). These measures were then entered into the Jamovi tool (v.2.3.28) to perform the Mann–Whitney test and the Wilcoxon rank test to assess between-group differences and longitudinal changes within each group respectively. This approach allows for quantitative comparison of spatial consistency patterns across conditions while acknowledging that ${OM}_{th-w}$ values are inherently aggregated at the group level. Therefore, statistical inference is interpreted at the spatial (ROI) level rather than at the level of individual subjects.

## 3. Results

In the following sections, the results concerning the negative emotion stimuli fMRI contrast are extensively reported, while the results pertaining to the positive emotions are detailed in the Supplementary Materials.

### 3.1. Participants

Thirty healthy subjects (mean age ± sd in years = 27.1 ± 7.2; 21 females) performed two separate acquisitions with an average inter-scan interval of 4.6 months.

Thirteen pwMS (mean age ± sd in years = 47.2 ± 11.9; 10 females) performed a pre-treatment MRI examination, but only 11/13 repeated the acquisition after the treatment. Demographic characteristics and depression scales (i.e., HADS and HDRS), measured at baseline and after treatment, are reported in Table 1 for pwMS.

Depressive symptoms in pwMS were significantly reduced (*p* < 0.05) after the EMDR rehabilitation treatment, as detailed in a previous work [23].

### 3.2. GLM Random Effect Group Analysis Results in Healthy Control Subjects

**Figure 2:**
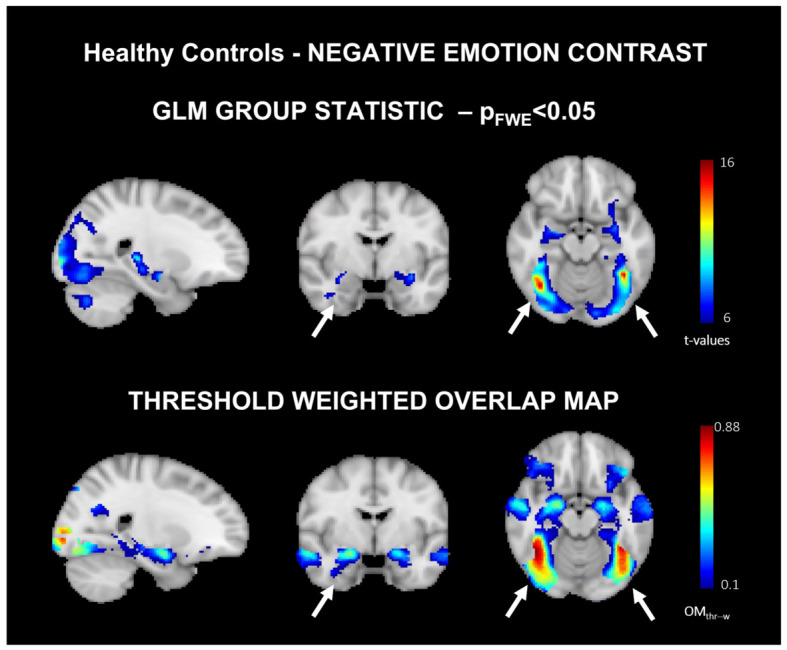
GLM mean group effects spatial maps (**top panel**) (pFWE < 0.05) and threshold weighted overlap maps (${OM}_{th-w},$ **bottom panel**) ([22]) derived at baseline for the negative emotion stimuli contrast. The maps are color-coded according to the t-values and consistency values for the GLM-derived maps and ${OM}_{th-w}$, respectively. Main differences between the two maps are indicated by white arrows.

Baseline: The main effect activation patterns of negative emotion stimuli are reported in Figure 2 (for positive emotions, refer to Figure S1) and in the top panel for HCs.

Longitudinal: No significant differences (pFWE < 0.05) were detected when comparing the first and second scans of the sub-sample of 30 subjects, using a paired *t*-test.

### 3.3. GLM Random Effect Group Analysis Results in People with Multiple Sclerosis

**Figure 3:**
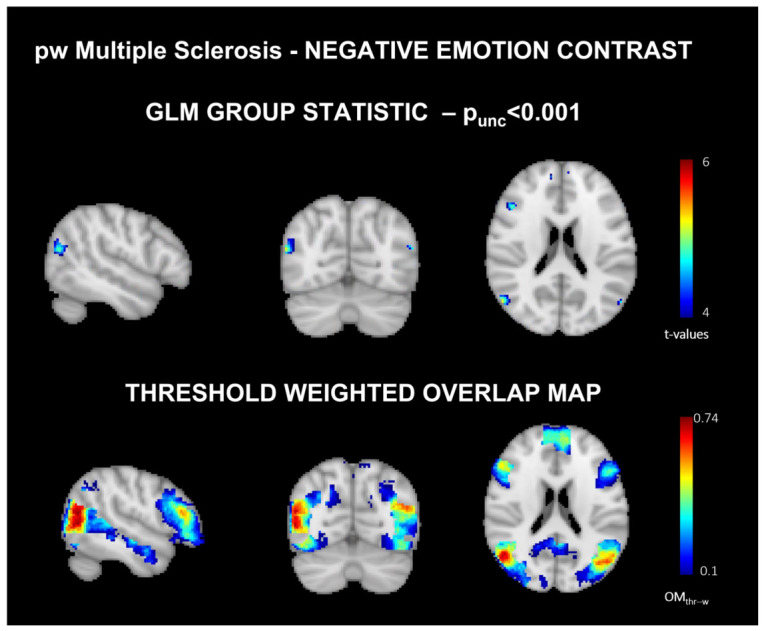
GLM mean group effect spatial maps (**top panel**) (p_unc_ < 0.001) and threshold-weighted overlap maps (${OM}_{th-w},$ **bottom panel**) ([22]) derived at baseline for the negative emotion stimuli contrast. The maps are color-coded according to the t-values and consistency values for the GLM-derived maps and ${OM}_{th-w}$, respectively.

Baseline: The main effect activation patterns of negative emotions stimuli are reported in Figure 3 (for positive emotions, refer to Figure S3) and the top panel for pwMS.

Longitudinal: No significant differences (pFWE < 0.05) emerged from the paired *t*-test comparing pwMS who underwent MRI evaluation pre- and post-rehabilitation treatment, as reported in [23].

### 3.4. Threshold-Weighted Overlap Map Results in Healthy Control Subjects

Baseline: Figure 2 ${OM}_{th-w}$ (bottom panel) for the negative emotion contrast. Of note, the activation of the amygdala is left-sided when looking at GLM-group effect (top-panel) while involved bilaterally when considering ${OM}_{th-w}$. Overall, the peak consistency value for negative emotions contrast-derived from ${OM}_{th-w}$ showed a decrement from 0.87 (maximum value) in the first scan to 0.79 in the second scan. The peak consistency value for positive emotion showed a similar decrement, going from a maximum value of 0.74 in the first scan to a maximum value of 0.64 in the second scan.

Longitudinal: Table 2 reports the delta values measuring the difference between the first and second scans in peak and average consistency for each ROI for the negative emotion stimuli (see Table S1 for positive emotion stimuli).

**Figure 4:**
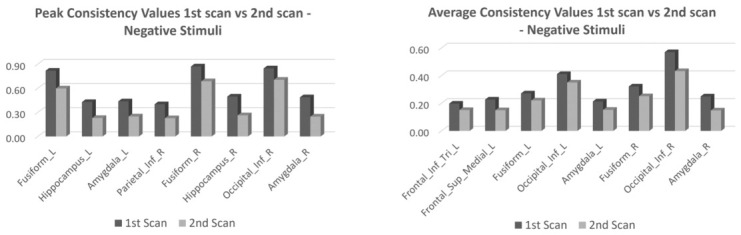
The bar charts represent the consistency peak (**left panel**) and average (**right panel**) values extracted from the ${OM}_{th-w}$ of negative stimuli for each ROI in the 1st and 2nd scans of the healthy control group. Legend: L = left; R = right; Sup = superior; Inf = inferior; Tri = triangular.

Figure 4 represents the bar charts of ROIs showing differences above the 75° percentile considering peak and mean values derived from the ${OM}_{th-w}$ for the negative (positive) contrast in the first and second scans in the control group.

Similar results were found concerning the positive stimuli (see Supplementary Materials: Figure S1, top panel; Figure S2; and Table S1).

A direct statistical comparison (Wilcoxon rank test) between the first and second scans, considering peak and average ${OM}_{th-w}$ values across ROIs, is reported in Table 3. Results showed significant differences (*p* < 0.001) for both positive and negative emotion contrasts with a large effect size.

### 3.5. Threshold-Weighted Overlap Map Results in People with Multiple Sclerosis

Baseline: Figure 3 shows (pre-EMDR) the ${OM}_{th-w}$ (bottom panel) for the negative emotion stimuli, with the latter providing a wider activation pattern compared to the first one (see Figure S3 for positive emotion stimuli). Moreover, Figure S5 shows that the peak consistency values for negative emotion contrast-derived from the ${OM}_{th-w}$ showed an increment from 0.70 pre-EMDR to 0.83 post-EMDR. For positive emotion, the peak consistency increased from 0.59 pre-EMDR to 0.78 post-EMDR.

Longitudinal: A general increment in consistency was observed in all ROI, as shown in Table 4, measuring the difference between pre- and post-rehabilitation scans in peak and average delta values for negative emotion stimuli.

**Figure 5:**
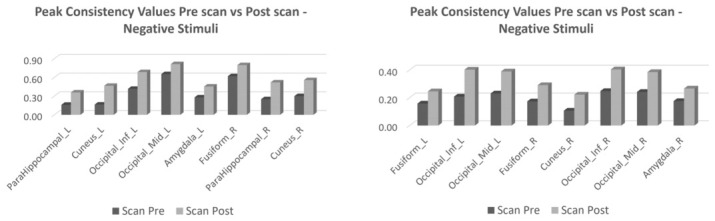
The bar charts represent the consistency peak (**left panel**) and average (**right panel**) values extracted from the ${OM}_{th-w}$ map of negative stimuli for each ROI of the pre- and post-rehabilitation scans of the pwMS. Legend: L = left; R = right; Mid = middle; Inf = inferior; Thal = thalamus.

Figure 5 represents the bar charts of ROIs showing differences above the 75° percentile, considering peak and mean values derived from ${OM}_{th-w}$ for negative emotion contrast in pre- and post-EMDR in the pwMS group.

Similar results were observed concerning the positive stimuli (see Supplementary Materials: Figure S3, top panel, Figure S4, and Table S2).

A direct statistical comparison (Wilcoxon rank test) between pre- and post-EMDR scans, considering peak and average ${OM}_{th-w}$ values across ROIs, is reported in Table 5. The results showed significant differences (*p* < 0.001) for both positive and negative emotion contrasts with a large effect size.

The comparison of ${OM}_{th-w}$, namely the spatial maps measuring consistency pre- and post-rehabilitation for the pwMS group, is reported in Figure S5.

### 3.6. Between-Group Comparison

Baseline: Table 6 reports a baseline comparison (Mann–Whitney test) of peak and average values extracted across ROIs from ${OM}_{th-w}$ showing significant differences between HCs and pwMS with a moderate effect size. Overall, the HC consistency was higher.

Longitudinal: The direct comparisons between delta values computed as differences between the peak and average values of the first (pre) vs. second (post) scan for the HC and pwMS groups, respectively, were significantly different (*p* < 0.001), with a large effect size when tested for mean differences using the Mann–Whitney test (Table 7). This was true for both positive and negative emotions contrast.

## 4. Discussion

This study aimed to evaluate the relevance of assessing the inter-subject variability during an emotion stimulation fMRI paradigm [24] for investigating neural changes in longitudinal settings. To this purpose, we evaluated a group of pwMS with depression undergoing EMDR rehabilitation treatment and a group of HCs using ${OM}_{th-w}$, [22], measuring the percentage of subjects sharing the neural activations across the statistical threshold.

The results presented herein suggest that ${OM}_{th-w}$ provides complementary information to standard GLM-based group analyses, capturing wider neural networks in both HC and pwMS. Furthermore, opposite longitudinal trends were observed in the two groups, with a decrease in consistency in HC and an increase in pwMS.

### 4.1. Task Consistency: Baseline Evaluation in Healthy Controls

The neural activation patterns retrieved in the HC group, considering the GLM standard group effects for both positive and negative emotions, are in line with a recent review [33], exploring large-scale brain networks underlying emotion generation, perception, and regulation.

${OM}_{th-w}$ revealed spatially wider activation clusters compared to GLM-derived maps, including the bilateral involvement of the amygdala, a relevant area for emotional processing. In contrast, the GLM results showed activation primarily in the left amygdala. Accordingly, the left amygdala is reportedly more frequently activated compared to its homologue [34], specifically for negative emotions [35], suggesting stronger inter-subject variability in the latter.

### 4.2. Task Consistency: Longitudinal Evaluation in Healthy Controls

The GLM-based evaluation did not detect any longitudinal changes in line with [24,36,37,38]. In contrast, the evaluation of ${OM}_{th-w}$ highlighted a robust longitudinal decrement in consistency in both positive and negative valence conditions. The pattern was observed in almost all of the considered ROIs; however, the magnitude of the effect was not homogeneous, suggesting a certain degree of region-specific modulation. The overall decrease in consistency should be interpreted with caution, as the present design does not allow for causal inferences. While one possible explanation may involve changes in neural efficiency or adaptation across repeated sessions [24], alternative explanations such as residual practice effects, inter-session variability, or uncontrolled subject-level factors cannot be excluded. This effect has been observed by previous studies [24,36] on intra-subject variability across repeated measures. This trend, as markedly highlighted by the peak and average delta values, was predominantly observed in occipital–visual areas and hippocampus, with the addition of the amygdala specifically when negative emotions are considered. Greater individual variability is indeed expected in the long term regarding the amygdala engagement during emotional processing, as previously observed specifically for the right amygdala [39].

### 4.3. Task Consistency: Baseline Evaluation in People with Multiple Sclerosis

The GLM group effects results prevented us from observing any significant cluster of activation in the pwMS group, both for positive and negative emotion stimuli. This result is likely influenced by the limited statistical power associated with the small sample size (i.e., 13 subjects), particularly given the known heterogeneity of neural responses in clinical populations. However, when looking at the derived ${OM}_{th-w}$, the difference is striking: the employment of consistency maps allowed us to observe neural patterns of activation similar to the ones observed for HC [24,36,37,38], comprising both sensory areas devoted to the processing of the visual stimuli (i.e., occipital cortex), as well as regions devoted to emotional such as the temporal cortex (middle temporal gyrus) and inferior frontal cortex (middle and inferior frontal gyri). Nonetheless, as expected, the consistency was overall reduced for pwMS with respect to the HC group. These results reflect more heterogeneous activations for pwMS, which could also be ascribed to the presence of different patterns of WM lesions and have probably also prevented the retrieval of more robust group effects. In addition, the age difference between the HCs and pwMS should be considered to be a potential confounding factor, as age is known to influence both the spatial extent and variability of functional activation patterns [40]. Since the ${OM}_{th-w}$ approach does not allow us to derive subject-specific values, excluding the possibility of including age as a covariate, its contribution to the observed differences in consistency values cannot be entirely excluded.

However, these results are in line with the atypical and higher variable neural response to emotional stimuli previously observed for pwMS compared to healthy controls. In fact, scattered neural activation in response to emotional stimuli was observed for cognitively preserved pwMS, especially related to stimuli with positive valence [41].

### 4.4. Task Consistency: Longitudinal Evaluation in People with Multiple Sclerosis

Concerning the GLM-based statistics, no differences were retrieved between pre- and post-EMDR rehabilitation sessions, neither in the positive emotion contrasts nor in the negative emotion contrasts [23]. The employment of ${OM}_{th-w}$ to assess inter-subject activation consistency allowed us instead to observe robust longitudinal changes. Interestingly, when comparing the neural activation patterns of pwMS before and after the rehabilitation, an opposite trend was observed with respect to the HC group, both in terms of peak and average consistency delta values. This trend is visible for almost all of the considered ROIs; however, a region-dependent modulation cannot be excluded. The increase in consistency observed in the pwMS group after EMDR treatment might be ascribed to a restoration of more typical neural patterns associated with emotional processing, resulting in more coherent and homogenous brain activity. Interestingly, the regions showing greater differences before and after EMDR treatment, such as the parahippocampal cortex, hippocampus, and amygdala, represent putative targets specific to the EMDR intervention [42]. Of note, the restored consistency in bilateral amygdala activation in response to negative stimuli might be related to the significant reduction in depressive symptoms after the EMDR rehabilitation treatment, as described in [23]. This interpretation should be considered with caution. Given the absence of a clinical control group (e.g., pwMS not undergoing EMDR), it is not possible to disentangle treatment-specific effects from other factors, such as time and repeated exposure to the task [24]. Therefore, the observed longitudinal changes cannot be unequivocally attributed to the EMDR intervention and should rather be interpreted as preliminary evidence of neural modulation occurring over time in this cohort.

### 4.5. Statistical Comparisons of ROI-Level ${OM}_{th-w}$ Measures

In addition to the qualitative assessment of spatial consistency patterns, ROI-based statistical comparisons were performed on ${OM}_{th-w}$-derived summary measures. Within-group longitudinal analyses revealed significant differences between pre- and post-treatment scans in pwMS, as well as between the first and second scans in HCs, for both peak and mean values across emotional conditions. Similarly, between-group comparisons showed significant differences in both baseline consistency values and longitudinal changes (delta values), with generally higher consistency observed in HCs compared to pwMS. These findings suggest systematic differences in spatial consistency between groups and across time. However, these results should be interpreted in an exploratory framework, as ROI-level measures derived from ${OM}_{th-w}$ represent aggregated indices of group consistency rather than independent subject-level observations. Consequently, statistical comparisons reflect differences in spatial summaries across predefined regions rather than fully independent regional tests. Overall, these quantitative results are consistent with the descriptive ${OM}_{th-w}$ maps, supporting the presence of group differences and longitudinal modulation in spatial consistency.

The present study is not exempt from limitations: this is a preliminary work aimed at assessing the degree of variability characterizing an emotion stimulation fMRI task paradigm in a straightforward and easy-to-interpret way. An important limitation concerns the small sample size, particularly referring to the longitudinal pwMS cohort (n = 11). According to the literature [43], with this sample size, statistical comparison can only detect large effects, while smaller but potentially meaningful effects are likely to remain undetected. This limited statistical power may explain the absence of significant findings in standard GLM-based analyses and suggests a non-negligible risk of false negatives. In this context, the use of ${OM}_{th-w}$ should be interpreted as an exploratory and complementary rather than alternative approach to standard inferential statistics, aimed at providing additional information when dealing with small sample sizes. Another limitation concerns the interpretation of the observed changes in spatial consistency, which may be influenced by non-neural factors, particularly in clinical populations such as pwMS. These include potential differences in image coregistration and normalization accuracy, residual head motion, and disease-related structural alterations, such as white matter lesions and brain atrophy, which may affect both signal quality and spatial alignment across subjects. Moreover, subject-level variables including age, baseline symptom severity, pharmacological treatment, and possible comorbidities may contribute to inter-subject variability and to the observed longitudinal effects. Although standard preprocessing pipelines and quality control procedures were applied to minimize these sources of variability, their influence cannot be entirely ruled out and should be systematically addressed in future studies with larger samples and more controlled designs. Future work is needed to implement multilevel models overcoming the limitation of GLM-based analysis in assessing between-individual differences of change over time [44].

## 5. Conclusions

Overall, the assessment of inter-subject variability revealed effects not captured by standard GLM group analyses [8,12,22,45,46]. Rather than providing definitive evidence of treatment effects, these findings primarily support a methodological contribution, highlighting the added value of explicitly modelling individual variability in longitudinal fMRI data.

This approach allowed the identification of meaningful activation patterns emerging in subgroups of participants (e.g., right amygdala in healthy controls), which may guide more targeted post hoc analyses, particularly in small or clinically heterogeneous samples where group-level effects are often underpowered. However, these results should be interpreted cautiously, as they may be influenced by confounding factors such as age differences and disease-related structural variability.

In clinical populations, characterizing typical and variable activation patterns may help distinguish pathological activity and better interpret longitudinal changes. In this context, variability assessment appears particularly relevant for capturing recovery-related mechanisms, as suggested by the observed normalization of amygdala activity in patients, consistent with previous findings and reduction in depressive symptoms following EMDR [23].

In conclusion, this pilot study supports the integration of inter-subject variability analyses with standard GLM approaches, especially in longitudinal designs where individual differences in recovery strategies are likely substantial and informative.

## Figures and Tables

**Table 1:** Demographic characteristics and clinical depression scales are reported in the table for pwMS. Statistical comparisons are computed using a paired *t*-test on Jamovi.

Group (N)	MS (13)		
**Age in years, mean ± SD**	47.2 ± 11.9 10 (77%)		
**Females, number (%)**			
**EDSS** **Mean ± SD**		3.32 ± 2.22	
**HADS** **mean ± SD**	Pre *	Post *	***p*** -values *
	9.27 ± 2.57	5.45 ± 4.32	0.009
**HDRS** **mean ± SD**	23.3 ± 6.08	7.7 ± 5.7	<0.001

Legend: HADS = Hospitalized Anxiety and Depression Scale; HDRS = Hamilton Depression Rating Scale; scoring marked with * are referred to the subsample of 11 pwMS who underwent EMDR treatment.

**Table 2:** Peak and average consistency values obtained from the ${OM}_{th-w}$ of the negative emotion stimuli in the HC group are reported. The delta values computed as differences between the 1st and 2nd scans are also reported for peak and average consistency. Delta values highlighted in bold refer to differences equal to or above the 75° percentile.

ROIs Labels	ROIs Number	Peak Consistency 1st Scan	Peak Consistency 2nd Scan	Delta Peak Consistency (1st Scan–2nd Scan)	Average Consistency 1st Scan	Average Consistency 2nd Scan	Delta Average Consistency (1st Scan–2nd Scan)
Frontal_Inf_Oper_L	1	0.33	0.28	0.05	0.11	0.09	0.02
Frontal_Inf_Orb_L	2	0.43	0.37	0.06	0.15	0.13	0.02
Frontal_Inf_Tri_L	3	0.41	0.37	0.04	0.20	0.15	**0.05**
Frontal_Sup_Medial_L	4	0.50	0.38	0.12	0.23	0.15	**0.08**
Insula_L	5	0.29	0.20	0.08	0.07	0.04	0.03
Parietal_Inf_L	6	0.27	0.27	−0.01	0.08	0.07	0.01
Precuneus_L	7	0.34	0.31	0.03	0.12	0.11	0.01
Fusiform_L	8	0.82	0.60	**0.22**	0.27	0.22	**0.05**
Hippocampus_L	9	0.43	0.23	**0.20**	0.13	0.11	0.03
ParaHippocampal_L	10	0.22	0.23	−0.01	0.05	0.07	−0.01
Temporal_Mid_L	11	0.79	0.71	0.08	0.22	0.19	0.03
Cuneus_L	12	0.32	0.24	0.08	0.11	0.09	0.02
Occipital_Inf_L	13	0.66	0.57	0.09	0.41	0.35	**0.06**
Occipital_Mid_L	14	0.80	0.75	0.04	0.34	0.33	0.01
Amygdala_L	15	0.44	0.25	**0.19**	0.21	0.15	**0.06**
Thal_L	16	0.24	0.23	0.01	0.06	0.05	0.01
Frontal_Inf_Oper_R	17	0.51	0.41	0.10	0.18	0.15	0.03
Frontal_Inf_Orb_R	18	0.32	0.31	0.02	0.14	0.12	0.02
Frontal_Inf_Tri_R	19	0.53	0.41	0.12	0.24	0.21	0.03
Frontal_Sup_Medial_R	20	0.47	0.37	0.10	0.19	0.15	0.04
Insula_R	21	0.20	0.19	0.01	0.05	0.04	0.00
Parietal_Inf_R	22	0.40	0.23	**0.17**	0.09	0.06	0.03
Precuneus_R	23	0.36	0.31	0.05	0.14	0.11	0.03
Fusiform_R	24	0.87	0.69	**0.18**	0.32	0.25	**0.07**
Hippocampus_R	25	0.50	0.26	**0.23**	0.17	0.12	0.05
ParaHippocampal_R	26	0.39	0.31	0.08	0.08	0.09	−0.01
Temporal_Mid_R	27	0.87	0.79	0.07	0.29	0.25	0.03
Cuneus_R	28	0.58	0.49	0.10	0.20	0.17	0.03
Occipital_Inf_R	29	0.85	0.71	**0.14**	0.57	0.43	**0.14**
Occipital_Mid_R	30	0.86	0.77	0.09	0.40	0.36	0.04
Amygdala_R	31	0.49	0.25	**0.24**	0.25	0.15	**0.10**
Thal_R	32	0.27	0.23	0.05	0.06	0.06	0.00

**Table 3:** First vs. second ${OM}_{th-w}$ value comparison across ROIs in HC group.

Stimuli Valence	${OM}_{th-w}$ Values (1st vs. 2nd)	*p*-Value	Effect Size (Rank Biserial Correlation)	Median Difference
Positive Emotions	Peak values	<0.001	0.89	0.077
	Average values	<0.001	0.85	0.030
Negative Emotions	Peak values	<0.001	0.99	0.090
	Average values	<0.001	1	0.105

**Table 4:** Peak and average Consistency values obtained from the ${OM}_{th-w}$ of the negative stimuli in the MS group are reported. The delta values computed as differences between the first and second scan are also reported for peak and average consistency. Delta values highlighted in bold refer to differences equal to or above the 75° percentile.

ROIs Labels	ROIs Number	Peak Consistency Scan Pre	Peak Consistency Scan Post	Delta Peak Consistency (Scan Pre–Scan Post)	Average Consistency Scan Pre	Average Consistency Scan Post	Delta Average Consistency (Scan Pre–Scan Post)
Frontal_Inf_Oper_L	1	0.30	0.35	−0.05	0.11	0.10	0.00
Frontal_Inf_Orb_L	2	0.32	0.30	0.02	0.13	0.11	0.03
Frontal_Inf_Tri_L	3	0.36	0.39	−0.03	0.15	0.16	−0.01
Frontal_Sup_Medial_L	4	0.49	0.45	0.04	0.19	0.16	0.03
Insula_L	5	0.25	0.32	−0.07	0.06	0.08	−0.01
Parietal_Inf_L	6	0.35	0.23	0.12	0.10	0.07	0.03
Precuneus_L	7	0.30	0.36	−0.05	0.10	0.12	−0.02
Fusiform_L	8	0.52	0.68	−0.16	0.16	0.25	**−0.09**
Hippocampus_L	9	0.22	0.37	−0.15	0.07	0.15	−0.08
ParaHippocampal_L	10	0.16	0.36	**−0.20**	0.06	0.12	−0.05
Temporal_Mid_L	11	0.64	0.80	−0.16	0.17	0.25	−0.08
Cuneus_L	12	0.16	0.47	**−0.30**	0.07	0.10	−0.03
Occipital_Inf_L	13	0.42	0.69	**−0.27**	0.21	0.40	**−0.19**
Occipital_Mid_L	14	0.65	0.81	**−0.16**	0.23	0.39	**−0.16**
Amygdala_L	15	0.28	0.45	**−0.17**	0.14	0.20	−0.07
Thal_L	16	0.16	0.29	−0.13	0.06	0.06	0.00
Frontal_Inf_Oper_R	17	0.40	0.40	0.00	0.14	0.13	0.00
Frontal_Inf_Orb_R	18	0.40	0.36	0.04	0.16	0.12	0.05
Frontal_Inf_Tri_R	19	0.49	0.47	0.02	0.20	0.19	0.01
Frontal_Sup_Medial_R	20	0.47	0.36	0.11	0.18	0.13	0.05
Insula_R	21	0.25	0.27	−0.02	0.07	0.08	−0.01
Parietal_Inf_R	22	0.32	0.45	−0.13	0.08	0.13	−0.05
Precuneus_R	23	0.29	0.42	−0.13	0.08	0.12	−0.04
Fusiform_R	24	0.62	0.80	**−0.18**	0.17	0.29	**−0.12**
Hippocampus_R	25	0.40	0.37	0.02	0.10	0.18	−0.08
ParaHippocampal_R	26	0.25	0.52	**−0.27**	0.08	0.16	−0.07
Temporal_Mid_R	27	0.70	0.83	−0.13	0.22	0.29	−0.07
Cuneus_R	28	0.30	0.56	**−0.26**	0.11	0.22	**−0.11**
Occipital_Inf_R	29	0.63	0.74	−0.11	0.25	0.40	**−0.16**
Occipital_Mid_R	30	0.62	0.75	−0.13	0.24	0.38	**−0.14**
Amygdala_R	31	0.40	0.45	−0.05	0.18	0.27	**−0.09**
Thal_R	32	0.27	0.25	0.02	0.08	0.06	0.02

**Table 5:** Pre- vs. post-EMDR ${OM}_{th-w}$ value comparison across ROIs in pwMS group.

Stimuli Valence	${OM}_{th-w}$ Values (Pre- vs. Post-EMDR)	*p*-Value	Effect Size (Rank Biserial Correlation)	Median Difference
Positive Emotions	Peak values	<0.001	−0.92	−0.105
	Average values	<0.001	−0.95	−0.045
Negative Emotions	Peak values	<0.001	−0.77	−0.095
	Average values	<0.001	−0.69	−0.045

**Table 6:** Between-group (HC vs. pwMS) comparison of peak and average values extracted across ROIs from baseline ${OM}_{th-w}$.

Stimuli Valence	${OM}_{th-w}$ Values (HC vs. pwMS)	*p*-Value	Effect Size (Rank Biserial Correlation)	Median Difference
Positive Emotions	ROIs Peak 1st/Pre Scan	0.029	0.32	0.079
	ROIs Avg 1st/Pre Scan	0.002	0.45	0.037
Negative Emotions	ROIs Peak 1st/Pre Scan	0.041	0.29	0.089
	ROIs Avg 1st/Pre Scan	0.077	0.26	0.038

**Table 7:** Between-group (HCs vs. pwMS) comparison of peak and average delta values (scan2–scan1) extracted across ROIs from ${OM}_{th-w}$.

Stimuli Valence	${OM}_{th-w}$ Delta Values (HCs vs. pwMS)	*p*-Value	Effect Size (Rank Biserial Correlation)	Median Difference
Positive Emotions	ROIs Peak	<0.001	0.91	0.1800
	ROIs Avg	<0.001	0.92	0.07
Negative Emotions	ROIs Peak	<0.001	0.85	0.19
	ROIs Avg	<0.001	0.76	0.0760

## Data Availability

The data presented in this study are available on request from the corresponding author due to privacy restriction.

## Supplementary Materials

The following supporting information can be downloaded at https://www.mdpi.com/article/10.3390/jimaging12050210/s1: **Figure S1**: Mean group effects spatial maps (top panel) derived from GLM standard statistic (p_FWE_ < 0.05) and consistency maps (bottom panel) threshold weighted overlap maps (${OM}_{th-w}$) derived according to [22] for the positive stimuli contrast of the healthy control group. The maps are color-coded according to the t-values and consistency values for the GLM derived maps and ${OM}_{th-w}$, respectively. Main differences between the two maps are pointed by white arrows; **Table S1**: Peak and Average Consistency values obtained from the ${OM}_{th-w}$ of the positive stimuli in HC group are reported. The delta values computed as differences between 1st and 2nd scan are also reported for peak and average consistency. Delta values highlighted in bold refers to differences equal or above the 75° percentile; **Figure S2**: The bar charts represent the consistency peak (left panel) and average (right panel) values extracted from the ${OM}_{th-w}$ map of positive stimuli for each ROIs of the 1st and 2nd scans of the healthy control group. Legend: L = Left; R = Right; Mid = Middle; Inf = Inferior; Thal = Thalamus; **Figure S3**: Comparison between mean group effects spatial maps (top panel) derived from GLM standard statistic (p_FWE_ < 0.05) and consistency maps (bottom panel) threshold weighted overlap maps (${OM}_{th-w}$) derived according to [22] for the positive stimuli contrast of the multiple sclerosis cohort. The maps are color-coded according to the t-values and consistency values for the GLM derived maps and ${OM}_{th-w}$ respectively; **Table S2**: Peak and Average Consistency values obtained from the ${OM}_{th-w}$ of the positive stimuli in MS group are reported. The delta values computed as differences between pre and post scan are also reported for peak and average consistency. Delta values highlighted in bold refers to differences equal or above the 75° percentile; **Figure S4**: The bar charts represent the consistency peak (left panel) and average (right panel) values extracted from the ${OM}_{th-w}$ map of positive stimuli for each ROIs of the pre and post-rehabilitation scans of the pwMS. Legend: L = Left; R = Right; Mid = Middle; Inf = Inferior; Thal = Thalamus; **Figure S5**: Comparison between pre (top panel) and post (bottom panel) rehabilitation ${OM}_{th-w}$ consistency maps derived for positive (left) and negative (right) stimuli contrasts in the pwMS group.

## Abbreviations

The following abbreviations are used in this manuscript:

HC	Healthy Controls
pwMS	People with multiple sclerosis
${OM}_{th-w}$	Threshold-weighted overlap maps
EMDR	Eye movement desensitization and reprocessing
