# Supplementary material for: Beyond GLM: Inter-Subject Variability as a Complementary Approach to Detect Longitudinal Changes in Emotion Processing in Multiple Sclerosis
Source: J Imaging. 2026 May 15;12(5):210. doi: 10.3390/jimaging12050210 (PMC13207876; doi:10.3390/jimaging12050210)
Supplement: Supplementary file 1 [file jimaging-12-00210-s001.zip › jimaging-4263900-supplementary/jimaging-4263900-supplementary_DEF.pdf]

Supplementary Materials

Results

GLM Random Effect Group Analysis Results

Healthy Control Subjects

At the group level, the neural activations for the positive emotions contrast were mainly located in bilateral middle and inferior occipital cortices, bilateral fusiform gyri, bilateral inferior frontal cortices (pars triangularis, and opercularis), bilateral middle temporal cortices, bilateral thalamus, left precentral cortex. The negative emotions contrast showed wider neural activation also comprising the right inferior temporal cortex.

People with Multiple Sclerosis

No suprathreshold ($p_{FWE}<0.05$) neural activity was observed for pwMS considering the acquisition performed on the whole sample preceding the EMDR treatment.

Threshold-weighted Overlap Maps Results

Healthy Control Subjects

The OM_{th-w} at the 1st resulted in 0.74 peak consistency for positive emotions contrast. The resulting consistency peak value for the 2nd was 0.64.

Figure S1 shows the comparison between GLM group statistics and OM_{th-w} compared to the GLM standard group results for the 1st scan of negative emotion contrast.

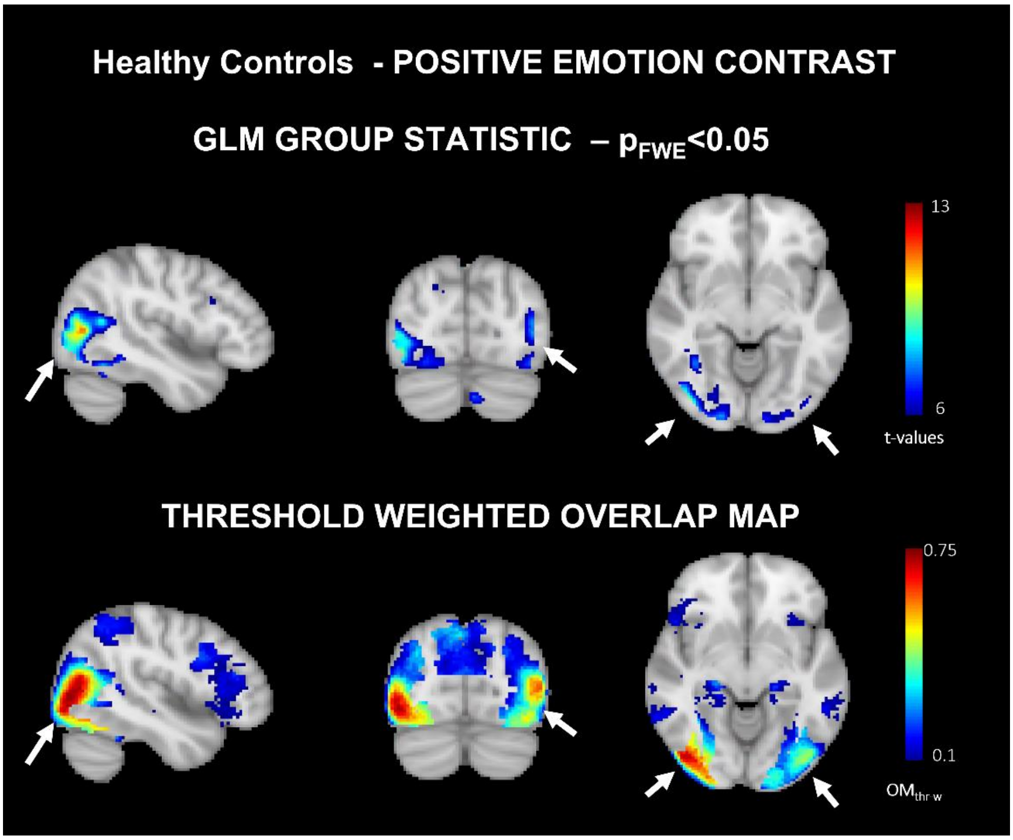

Figure S1. Mean group effects spatial maps (top-panel) derived from GLM standard statistic (p_{FWE}<0.05) and consistency maps (bottom-panel) threshold weighted overlap maps (OM_{th-w}) derived according to (Seghier & Price, 2016) for the positive stimuli contrast of the healthy control group. The maps are color-coded according to the t-values and consistency values for the GLM derived maps and OM_{th-w}, respectively. Main differences between the two maps are pointed by white arrows.

Table S1 reports the delta values measuring the difference between the 1st and 2nd in peak and average consistency for each ROI for positive and negative stimuli respectively.

Table S1. Peak and Average Consistency values obtained from the $OM_{th-w}$of the positive stimuli in HC group are reported. The delta values computed as differences between 1st and 2nd scan are also reported for peak and average consistency. Delta values highlighted in bold refers to differences equal or above the 75° percentile.

ROIs Labels	ROIs Number	Peak Consistency 1st Scan	Peak Consistency 2nd Scan	Delta Peak Consistency	Average Consistency 1st Scan	Average Consistency 2nd Scan	Delta Average Consistency

				(1st Scan – 2nd Scan)			(1st Scan – 2nd Scan)
Frontal_Inf_Oper_L	1	0.29	0.25	0.04	0.10	0.07	0.03
Frontal_Inf_Orb_L	2	0.15	0.23	-0.08	0.06	0.11	-0.04
Frontal_Inf_Tri_L	3	0.25	0.25	0.00	0.11	0.10	0.01
Frontal_Sup_Medial_L	4	0.23	0.27	-0.03	0.10	0.11	0.00
Insula_L	5	0.19	0.21	-0.03	0.07	0.05	0.02
Parietal_Inf_L	6	0.29	0.24	0.06	0.13	0.08	0.04
Precuneus_L	7	0.30	0.25	0.05	0.12	0.11	0.01
Fusiform_L	8	0.62	0.45	**0.17**	0.21	0.14	**0.07**
Hippocampus_L	9	0.22	0.16	0.06	0.08	0.07	0.01
ParaHippocampal_L	10	0.16	0.11	0.05	0.05	0.04	0.01
Temporal_Mid_L	11	0.63	0.47	**0.16**	0.12	0.12	0.00
Cuneus_L	12	0.37	0.23	**0.14**	0.18	0.11	**0.07**
Occipital_Inf_L	13	0.58	0.46	0.12	0.32	0.26	**0.07**
Occipital_Mid_L	14	0.65	0.54	0.11	0.24	0.20	0.04
Amygdala_L	15	0.20	0.14	0.06	0.09	0.07	0.02
Thal_L	16	0.22	0.18	0.05	0.10	0.06	0.04
Frontal_Inf_Oper_R	17	0.25	0.21	0.04	0.10	0.08	0.03
Frontal_Inf_Orb_R	18	0.19	0.16	0.03	0.06	0.06	0.00
Frontal_Inf_Tri_R	19	0.27	0.23	0.03	0.10	0.08	0.02
Frontal_Sup_Medial_R	20	0.22	0.23	-0.01	0.07	0.09	-0.01
Insula_R	21	0.17	0.11	0.06	0.06	0.04	0.02
Parietal_Inf_R	22	0.27	0.18	0.10	0.14	0.08	**0.06**
Precuneus_R	23	0.33	0.25	0.08	0.16	0.12	0.04
Fusiform_R	24	0.69	0.55	0.14	0.26	0.19	**0.07**
Hippocampus_R	25	0.29	0.15	**0.14**	0.10	0.08	0.02
ParaHippocampal_R	26	0.27	0.19	0.08	0.06	0.05	0.01
Temporal_Mid_R	27	0.73	0.64	0.09	0.18	0.17	0.00
Cuneus_R	28	0.50	0.33	**0.17**	0.24	0.14	**0.10**
Occipital_Inf_R	29	0.73	0.58	**0.15**	0.47	0.39	**0.08**
Occipital_Mid_R	30	0.74	0.58	**0.16**	0.26	0.21	**0.06**
Amygdala_R	31	0.18	0.13	0.05	0.09	0.07	0.02
Thal_R	32	0.31	0.17	**0.14**	0.09	0.06	0.03

Figure S2 represents the bar charts of ROIs showing differences above the 75° percentile considering peak and mean values derived from the OM_{th-w} of the positive emotion contrast in the 1st and in the 2nd scans in the control group.

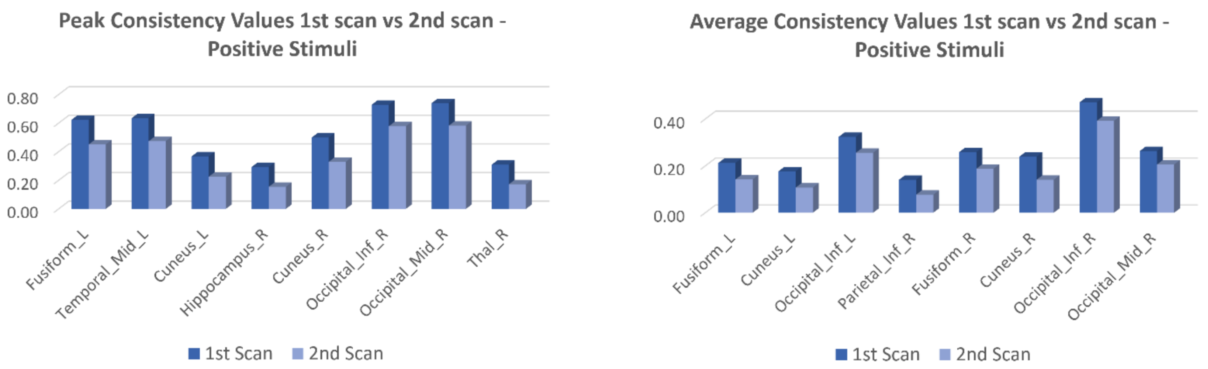

Figure S2. The bar charts represent the consistency peak (left panel) and average (right panel) values extracted from the OM_{th-w} map of positive stimuli for each ROIs of the 1st and 2nd scans of the healthy control group. Legend: L = Left; R = Right; Mid = Middle; Inf = Inferior; Thal = Thalamus.

People with Multiple Sclerosis

The OM_{th-w} pre-rehabilitation resulted in 0.59 peak consistency for positive emotion contrast. The resulting consistency peak value post-rehabilitation was 0.78.

Figure S3 shows the comparison between GLM group statistics and OM_{th-w} compared to the GLM standard group results for the pre-rehabilitation scan of positive stimuli contrast.

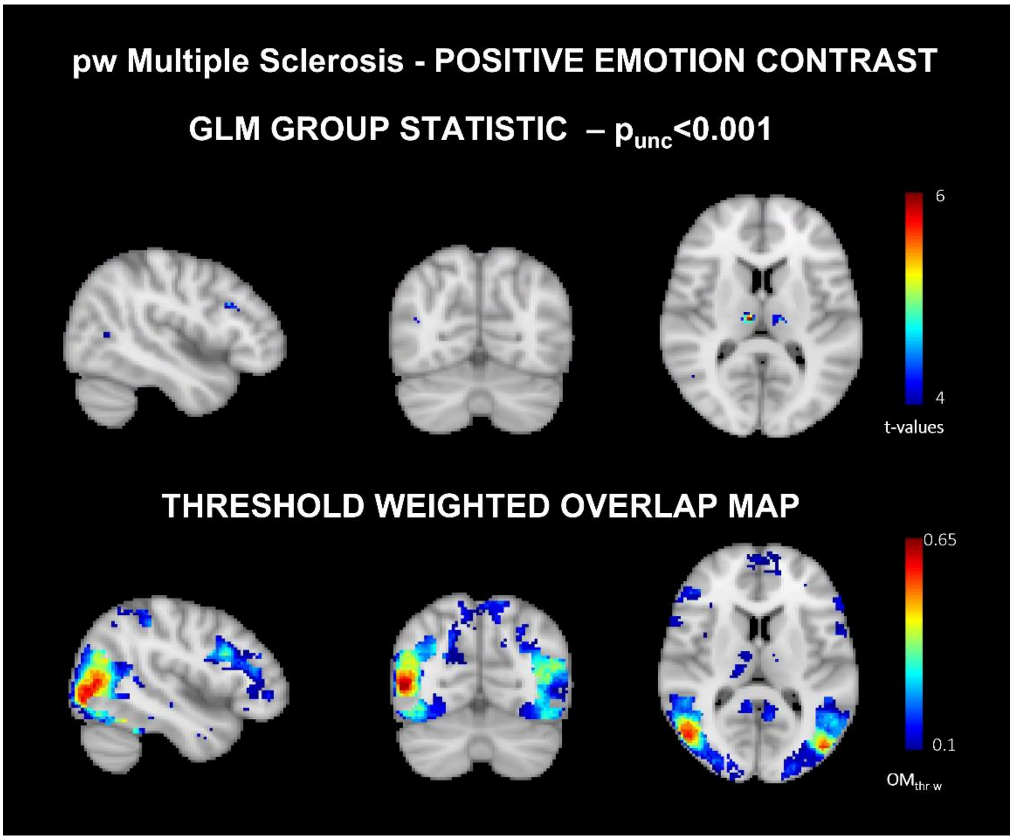

Figure S3. Comparison between mean group effects spatial maps (top-panel) derived from GLM standard statistic (p_{FWE}<0.05) and consistency maps (bottom-panel) threshold weighted overlap maps (OM_{th-w}) derived according to (Seghier & Price, 2016) for the

positive stimuli contrast of the multiple sclerosis cohort. The maps are color-coded according to the t-values and consistency values for the GLM derived maps and OM_{th-w} respectively.

Table S2. Peak and Average Consistency values obtained from the OM_{th-w} of the positive stimuli in MS group are reported. The delta values computed as differences between pre and post scan are also reported for peak and average consistency. Delta values highlighted in bold refers to differences equal or above the 75° percentile.

ROIs Labels	ROIs Number	Peak Consistency Scan pre	Peak Consistency Scan post	Delta Peak Consistency (Scan pre – Scan post)	Average Consistency Scan pre	Average Consistency Scan post	Delta Average Consistency (Scan pre – Scan post)
Frontal_Inf_Oper_L	1	0.38	0.28	0.10	0.11	0.09	0.01
Frontal_Inf_Orb_L	2	0.13	0.22	-0.09	0.05	0.07	-0.02
Frontal_Inf_Tri_L	3	0.31	0.28	0.03	0.09	0.11	-0.02
Frontal_Sup_Medial_L	4	0.16	0.27	-0.10	0.06	0.10	-0.04
Insula_L	5	0.11	0.18	-0.07	0.04	0.07	-0.02
Parietal_Inf_L	6	0.20	0.25	-0.05	0.08	0.09	-0.01
Precuneus_L	7	0.21	0.32	-0.11	0.06	0.14	**-0.08**
Fusiform_L	8	0.37	0.52	-0.15	0.11	0.21	**-0.10**
Hippocampus_L	9	0.07	0.22	-0.14	0.04	0.09	-0.05
ParaHippocampal_L	10	0.09	0.25	**-0.16**	0.04	0.07	-0.02
Temporal_Mid_L	11	0.42	0.59	**-0.17**	0.10	0.14	-0.05
Cuneus_L	12	0.14	0.23	-0.09	0.05	0.11	-0.06
Occipital_Inf_L	13	0.37	0.54	**-0.17**	0.16	0.34	**-0.18**
Occipital_Mid_L	14	0.56	0.63	-0.07	0.15	0.28	**-0.13**
Amygdala_L	15	0.14	0.21	-0.07	0.06	0.09	-0.03
Thal_L	16	0.14	0.16	-0.02	0.05	0.06	-0.02
Frontal_Inf_Oper_R	17	0.32	0.30	0.01	0.13	0.12	0.01
Frontal_Inf_Orb_R	18	0.21	0.23	-0.02	0.06	0.08	-0.01
Frontal_Inf_Tri_R	19	0.28	0.39	-0.11	0.10	0.12	-0.02
Frontal_Sup_Medial_R	20	0.20	0.23	-0.03	0.06	0.08	-0.02
Insula_R	21	0.14	0.23	-0.09	0.05	0.08	-0.03
Parietal_Inf_R	22	0.25	0.41	**-0.17**	0.09	0.09	0.00
Precuneus_R	23	0.27	0.38	-0.11	0.07	0.11	-0.04
Fusiform_R	24	0.58	0.59	-0.02	0.16	0.23	**-0.08**
Hippocampus_R	25	0.11	0.29	**-0.18**	0.04	0.11	-0.06
ParaHippocampal_R	26	0.16	0.32	-0.16	0.05	0.07	-0.02
Temporal_Mid_R	27	0.59	0.78	**-0.19**	0.17	0.21	-0.04
Cuneus_R	28	0.33	0.45	-0.12	0.09	0.23	**-0.14**
Occipital_Inf_R	29	0.50	0.74	**-0.24**	0.19	0.38	**-0.19**
Occipital_Mid_R	30	0.55	0.72	**-0.18**	0.19	0.30	**-0.11**
Amygdala_R	31	0.11	0.25	-0.14	0.06	0.12	-0.06
Thal_R	32	0.11	0.23	-0.12	0.04	0.06	-0.01

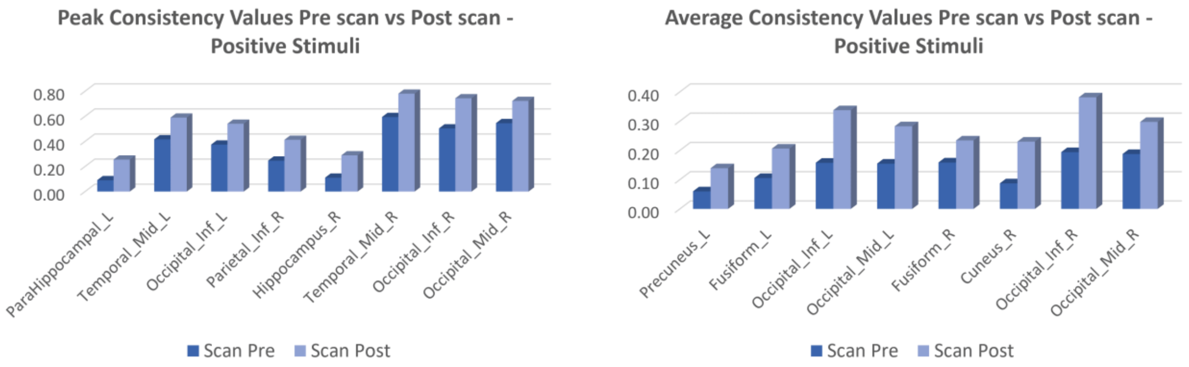

Figure S4. The bar charts represent the consistency peak (left panel) and average (right panel) values extracted from the OM_{th-w} map of positive stimuli for each ROIs of the pre and post-rehabilitation scans of the pwMS. Legend: L = Left; R = Right; Mid = Middle; Inf = Inferior; Thal = Thalamus.

The comparison of the OM_{th-w}, namely the spatial maps measuring consistency independently from the statistical thresholds pre- and post-rehabilitation for the MS group is reported in Figure S5.

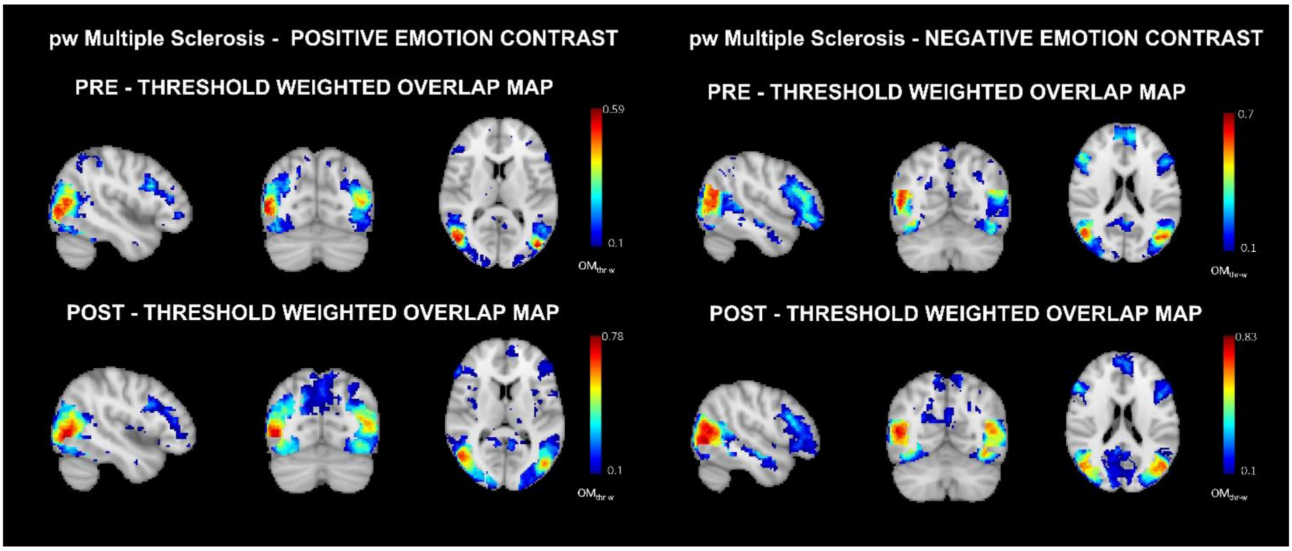

Figure S5. Comparison between pre (top-panel) and post (bottom-panel) rehabilitation OM_{th-w} consistency maps derived for positive (left) and negative (right) stimuli contrasts in the pwMS group.
