# Supplementary figures and images for: Beyond GLM: Inter-Subject Variability as a Complementary Approach to Detect Longitudinal Changes in Emotion Processing in Multiple Sclerosis
Source: J Imaging. 2026 May 15;12(5):210. doi: 10.3390/jimaging12050210 (PMC13207876; doi:10.3390/jimaging12050210)

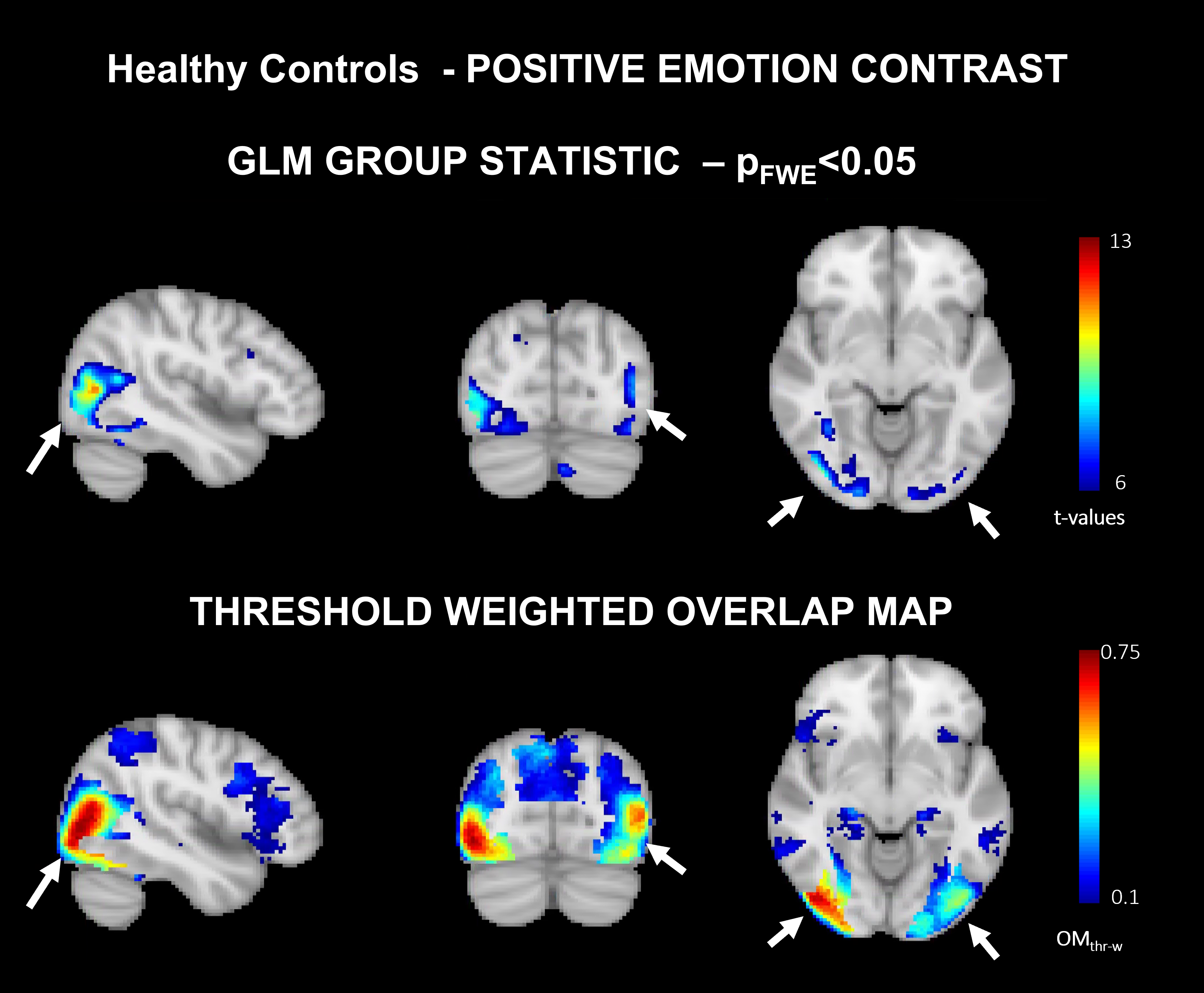

Supplement: Supplementary file 1 [file jimaging-12-00210-s001.zip › jimaging-4263900-supplementary/Figure_S1_DEF.png]

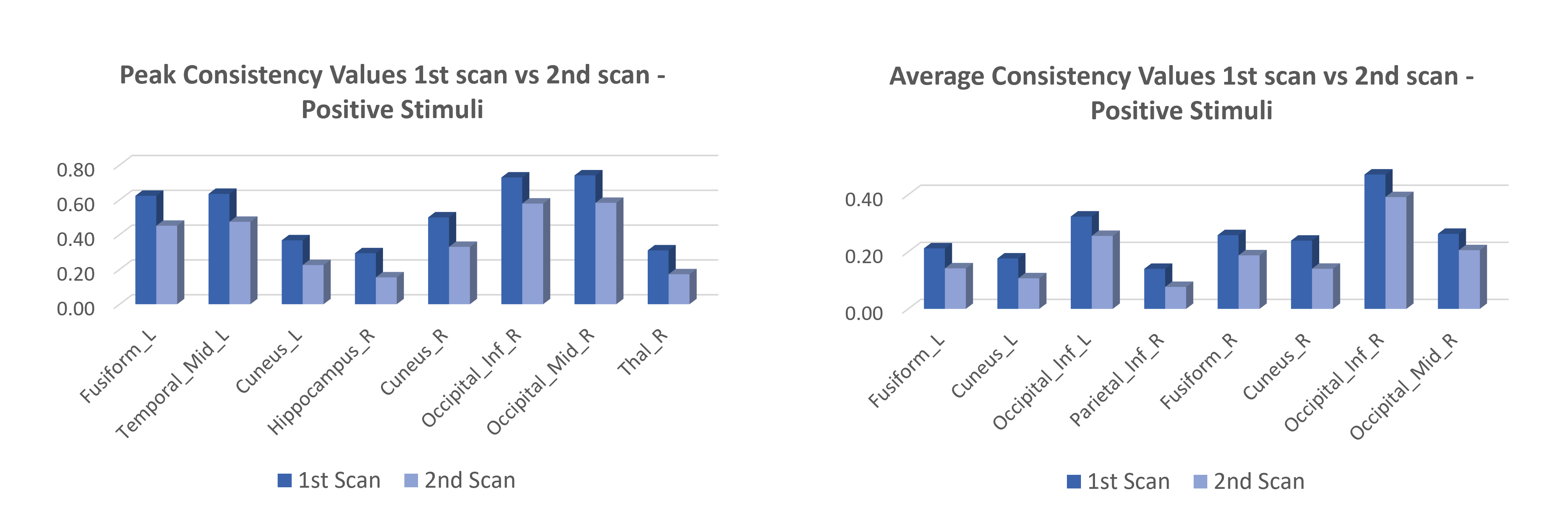

Supplement: Supplementary file 1 [file jimaging-12-00210-s001.zip › jimaging-4263900-supplementary/Figure_S2_DEF.png]

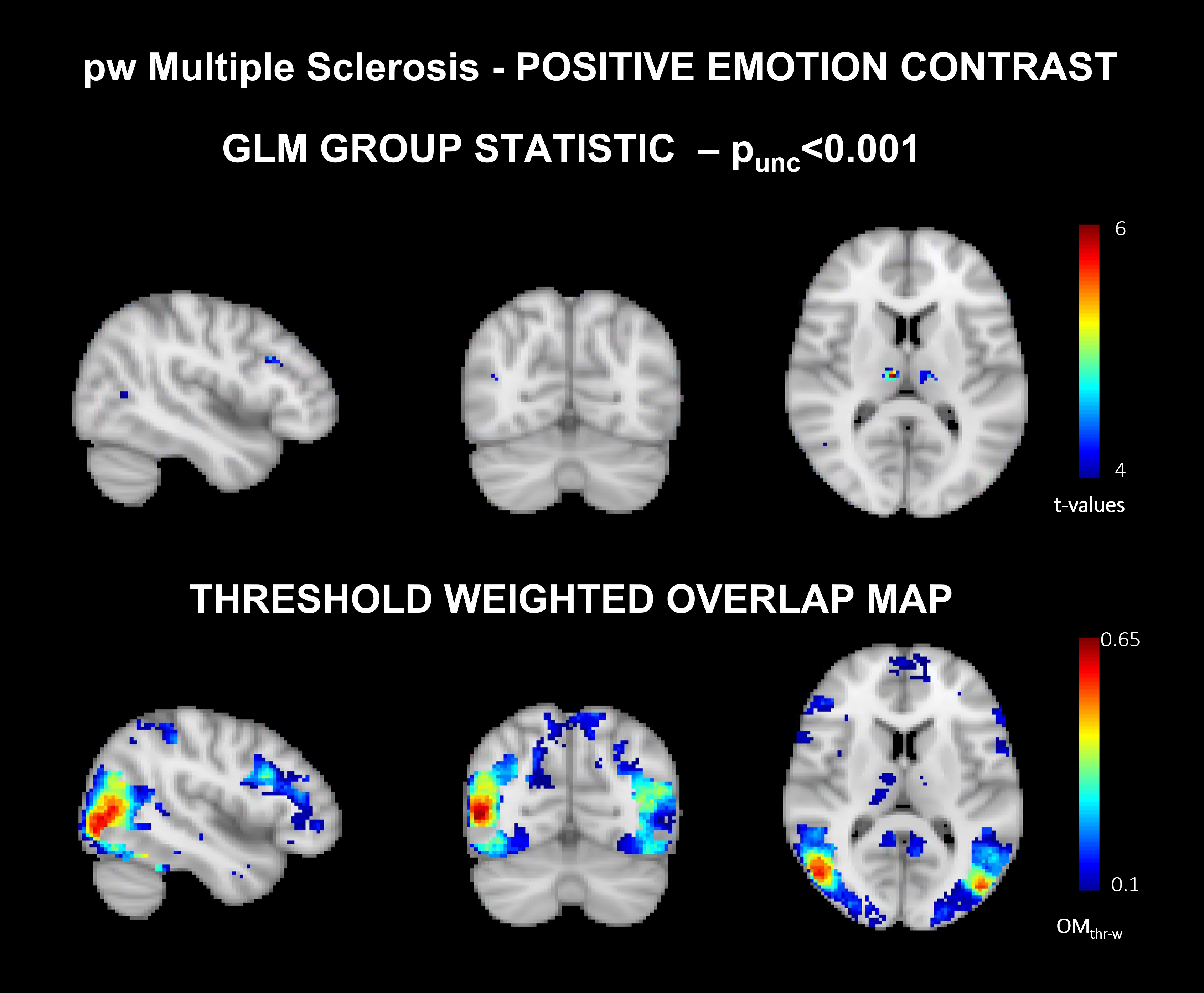

Supplement: Supplementary file 1 [file jimaging-12-00210-s001.zip › jimaging-4263900-supplementary/Figure_S3_DEF.png]

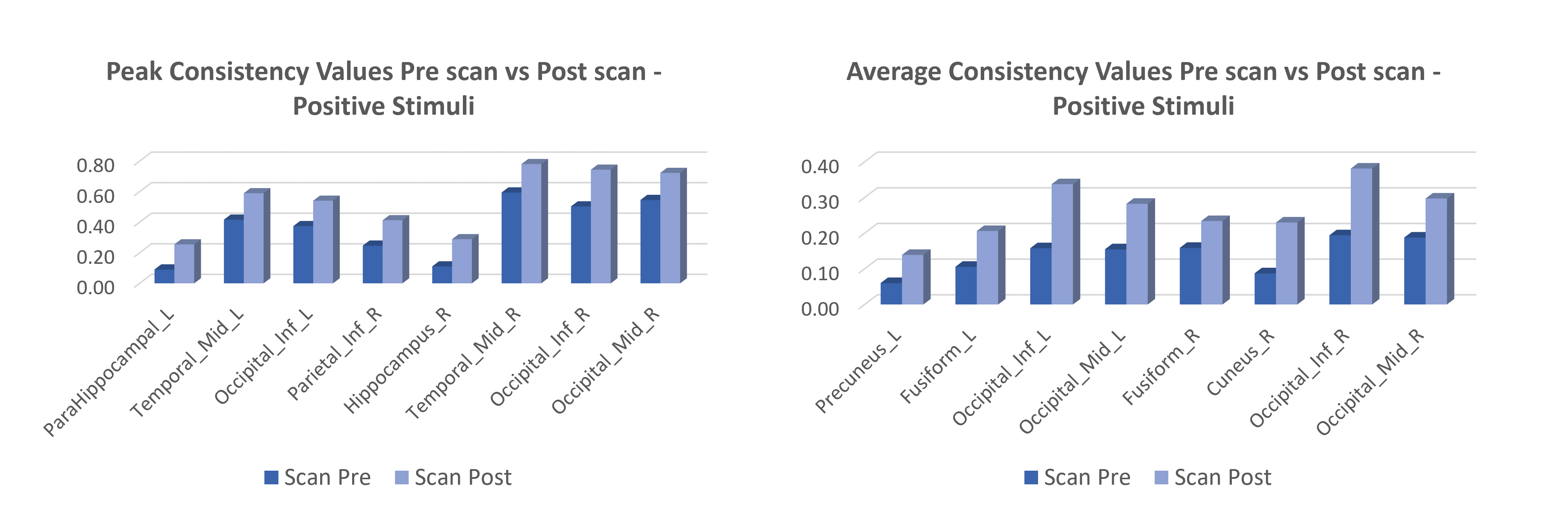

Supplement: Supplementary file 1 [file jimaging-12-00210-s001.zip › jimaging-4263900-supplementary/Figure_S4_DEF.png]

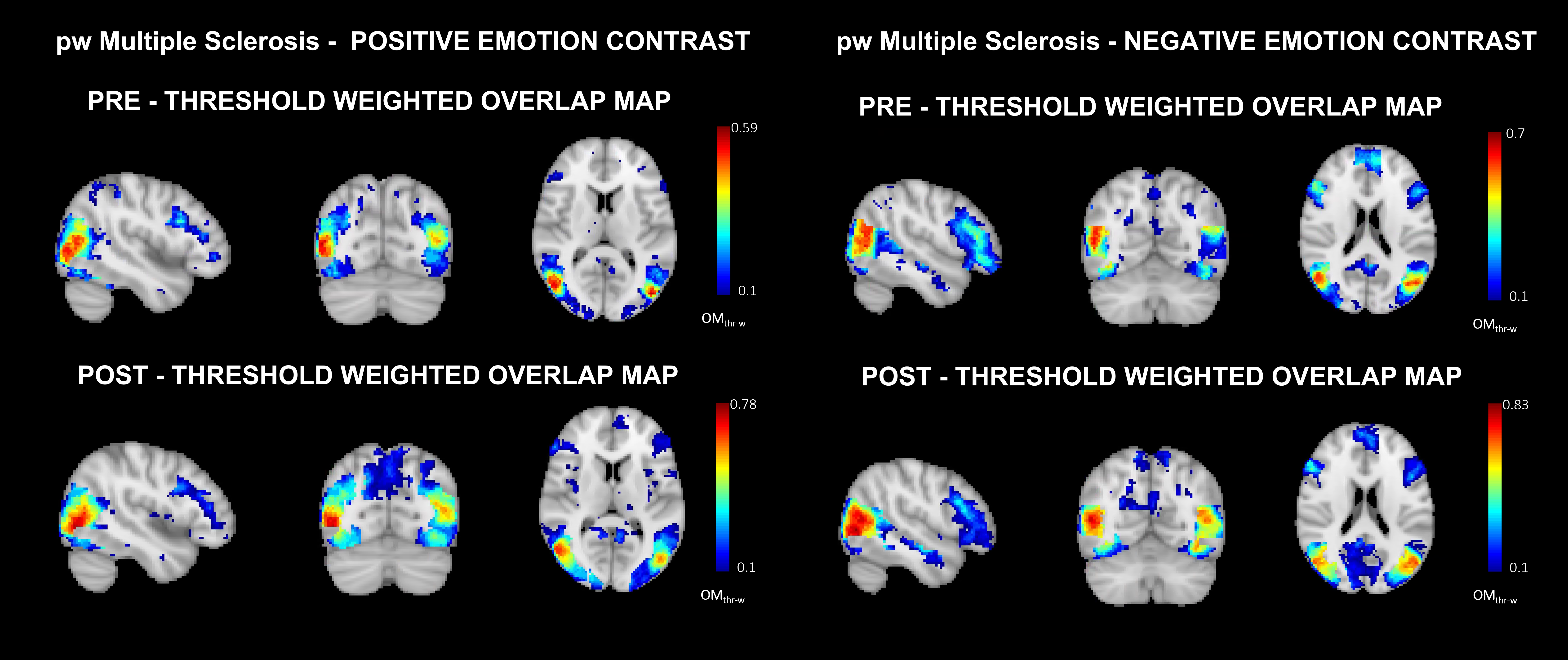

Supplement: Supplementary file 1 [file jimaging-12-00210-s001.zip › jimaging-4263900-supplementary/Figure_S5_DEF.png]
